# Wing shape differences between flying and non-flying individuals of six *Triatoma* species (Hemiptera: Reduviidae: Triatominae)

**DOI:** 10.1590/0037-8682-0276-2024

**Published:** 2025-03-17

**Authors:** Fernanda Cristina de Oliveira Firmino, Cleber Galvão, Dayse Rocha

**Affiliations:** 1 Instituto Oswaldo Cruz, Laboratório Nacional e Internacional de Referência em Taxonomia de Triatomíneos, Rio de Janeiro, RJ, Brasil.

**Keywords:** Triatoma, Wings, Shape, Flight ability, Chagas disease

## Abstract

**Background:**

Despite numerous reports of triatomines invading homes through flight, experimental studies investigating this ability under laboratory conditions remain scarce. Flight ability varies between individuals (males and females) of the same species, and wing shape modifications may be associated with the flight capacity of these vectors. Therefore, studies on this topic are crucial for the early identification of species with a greater propensity to invade human dwellings and for the implementation of appropriate surveillance and vector control strategies. However, even when exposed to the same nutritional and environmental conditions, triatomines demonstrate a range of flight abilities. To date, it remains unclear whether differences in wing morphology play a decisive role in determining this behavior. As an initial approach to addressing this issue, the present study aimed to determine whether morphometric differences exist in the wings of flying and non-flying individuals from six species of the genus *Triatoma*: *Triatoma costalimai*, *Triatoma klugi*, *Triatoma matogrossensis*, *Triatoma rubrovaria*, *Triatoma vandae*, and *Triatoma williami*.

**Methods:**

This study employed geometric morphometrics to investigate differences in wing size and shape between flying and non-flying individuals from six *Triatoma* species.

**Results:**

The findings indicated a variation in wing size between sexes. Additionally, shape variations were observed between flying and non-flying insects, particularly in females.

**Conclusions:**

Geometric morphometrics effectively identified distinct wing shape patterns in flying and non-flying specimens from six *Triatoma* species, revealing differences that may aid in identifying species with greater active dispersal capacity.

Triatomines are hematophagous insects that serve as vectors of *Trypanosoma cruzi* (Chagas, 1909), the etiological agent of Chagas disease, which is classified by the World Health Organization as a neglected tropical disease^1^. To date, 158 taxa have been described, 64 of which are found in Brazil, grouped into 18 genera and 5 tribes, with the vast majority occurring in the Americas^2^ ^,^ ^3^. All triatomine species are considered potential vectors of *T. cruzi*, which infects a wide range of wild and domestic mammalian species.

In Brazil, vector-borne transmission of Chagas disease by *Triatoma infestans* (Klug, 1834) has been officially eliminated since 2006, and efforts have been made to interrupt transmission by non-native vectors. Despite this significant achievement, certification of Chagas disease vector elimination may create the false impression that disease transmission no longer occurs, leading to the overlooking of native triatomine species that remain potential vectors of *T. cruzi* and that can invade and colonize domestic and peridomestic environments. The domiciliation of Triatominae is one of the key factors contributing to the increased risk of *T. cruzi* transmission to humans. Anthropogenic morphoclimatic alterations promote the domiciliation and dispersal of triatomines (e.g., through flight) and can be interpreted as a survival strategy for these species^2^. This is the case for the six Brazilian species examined in this study: *Triatoma costalimai* Verano & Galvão, 1958, *Triatoma klugi* Carcavallo, Jurberg, Lent & Galvão, 2001, *Triatoma matogrossensis* Leite & Barbosa, 1953, *Triatoma rubrovaria* (Blanchard, 1846), *Triatoma vandae* Carcavallo, Jurberg, Rocha, Galvão, Noireau & Lent, 2002, and *Triatoma williami* Galvão, Souza & Lima, 1965.

*T. costalimai* is a sylvatic species that frequently invades homes, and its presence and occurrence in domestic environments have been increasing since the 1990s. *T. costalimai* was first described from insects collected in calcareous soil in peridomestic environments and is associated with reptiles, rodents, and primates. This vector is found in the states of Bahia, Distrito Federal, Goiás, Mato Grosso, Mato Grosso do Sul, Minas Gerais, and Tocantins, its type locality^2^ ^,^ ^4^. 

*T. klugi* is found in Rio Grande do Sul. This species is sylvatic and inhabits cracks along cliff faces. It has been experimentally infected with both *T. cruzi* and *Trypanosoma rangeli* ^2^ *.*

*T. matogrossensis* is found in the state of Mato Grosso do Sul. It is predominantly a sylvatic species, although it can also be found in artificial environments^2^. 

*T. rubrovaria* is found in the state of Rio Grande do Sul. This wild species primarily inhabits exfoliated rocks but has increasingly been detected in peridomestic and domestic environments following the control of *T. infestans*. It may be a highly competent vector of *T. cruzi* ^2^ ^,^ ^7^. 

*T. vandae* is found in the states of Mato Grosso and Mato Grosso do Sul. It is a sylvatic species that is morphologically similar to *Triatoma jurbergi* Carcavallo, Galvão & Lent, 1998. This species was first observed on rocky walls without any known association with other triatomine species or potential hosts^2^. One year after its description, 193 insects were captured in peridomestic areas (in the state of Mato Grosso) and analyzed using the precipitin technique, which identified six distinct food sources: rodents, opossums, pigs, armadillos, dogs, and lizards^2^. 

*T. williami* is found in the states of Goiás, Mato Grosso, and Mato Grosso do Sul. This is a sylvatic species but has also been collected inside homes and artificial environments. It has been found naturally infected with *T. cruzi* ^5^.

All species analyzed are sylvatic, and except for *T. klugi*, all were identified in both intra- and peridomestic environments. These data suggest that the species may be moving between these environments, possibly through active or passive dispersal. Although there is still no concrete information on the dispersal strategies employed by these species, it is likely that flight is used by adults. Understanding the flight dynamics of these species is essential to mitigating the risks associated with Chagas disease transmission^6^.

In recent years, there have been increasing reports of sylvatic triatomine species invading human dwellings and peridomestic areas in several South American countries^6^. Most of these reports involve adult insects, and flight is considered a major means of dispersal in these invasions, likely constituting one of the primary mechanisms for triatomine infestation of homes^8^. 

Flight ability varies both among and within triatomine species, as well as between sexes and individuals. Thus, studies on flight ability may be useful for the early identification of species with a higher propensity to invade human dwellings, facilitating the implementation of appropriate vector surveillance measures. Despite numerous reports of triatomines invading homes through flight, experimental studies on flight behavior under controlled laboratory conditions remain scarce^8^. 

The active dispersal of adult triatomines via flight has been attributed by several authors to increases in ambient temperature and, more notably, to nutritional deficits. However, insects exposed to identical nutritional and temperature conditions exhibit variation in flight ability, and it remains unclear whether differences in wing morphology play a role in this behavior. As an initial approach to addressing this question, this study aimed to determine whether morphometric differences exist between the wings of flying and non-flying triatomines in six species of the genus *Triatoma* ^6^ ^,^ ^8^ ^,^ ^9^.

Geometric morphometrics is a cost-effective tool capable of identifying morphological shape patterns influenced by environmental and genetic factors. By analyzing differences in wing shape, it can detect variations in populations with low genetic diversity, making it crucial for identifying the origins of reinfestations^15^.

Sexual dimorphism in wing morphology is important not only for understanding the physiology and biomechanics of triatomine vectors but also for the development of entomological surveillance strategies. Models predicting vector migratory propensity, based on wing morphology, may serve as early-warning tools for assessing the risk of Chagas disease spread^14^.

Males and females from six closely related species of the genus *Triatoma* were used in this study (Table 1). *T. rubrovaria* and *T. klugi* are the most closely related to each other but belong to a sister clade that includes *T. costalimai*, *T. matogrossensis*, *T. vandae,* and *T. williami* ^9^. All species were collected from the southern and central-western regions of Brazil and reared in colonies at the insectary of the National and International Reference Laboratory in Taxonomy of Triatomines, Instituto Oswaldo Cruz (Rio de Janeiro). Species were classified based on their flight ability according to the procedure developed by Galvão et al.^10^. In this method, marked insects were placed in a "flight apparatus", which consisted of a 1-L beaker containing a vertical wooden rod in the center, allowing the insects to climb and attempt flight from the top. This jar was placed inside a plastic bucket measuring 80 cm in height and 50 cm in diameter, covered with a nylon screen.

**TABLE 1:** Number of individuals analyzed per species and flight trait in the genus *Triatoma*

Species	Trait			
	NF-F	F-F	NF-M	F-M
*T. costalimai*	3	10	0	6
*T. matogrossensis*	1	12	0	6
*T. vandae*	1	7	1	4
*T. williami*	0	5	0	1
*T. klugi*	3	0	1	0
*T. rubrovaria*	10	8	8	4
**Total**	**18**	**42**	**10**	**21**

**NF-F:** non-flying females; **F-F:** flying females; **NF-M:** non-flying males; **F-M:** flying males.

Since the insects were unable to escape from the glass jar by climbing its walls, those found in the bucket were classified as fliers, while those that remained in the jar were considered non-flying. Wingless insects were used as a control.

Wing measurements were obtained from landmark-based geometric morphometric data. The geometry of the morphological structures was captured by digitizing two-dimensional coordinates of biologically definable landmarks (Figure 1).

**FIGURE 1:**
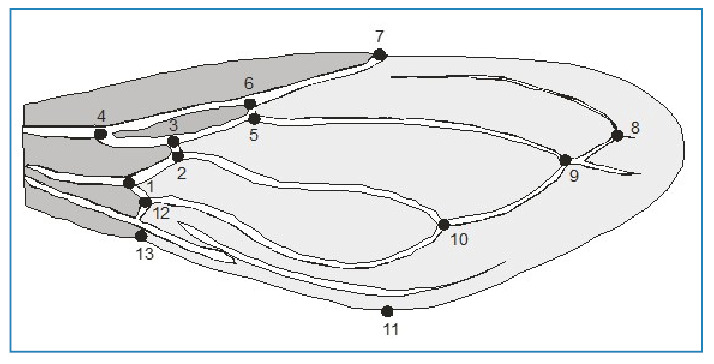
Wing from a *T. rubrovaria* specimen showing the landmarks used in this study to discriminate between flying and non-flying insects.

A generalized Procrustes analysis was applied to superimpose landmark configurations using the least-squares criterion, mathematically removing non-shape variation. Once non-shape variation was eliminated, the remaining variables represented pure shape data, which were used for statistical comparisons and graphical shape representations. Principal Component Analysis was employed to cluster and reduce the dataset. By analyzing the principal component axes, differences in shape and their directional changes between groups were identified based on deformation grids. Discriminant Analysis was conducted to generate Mahalanobis distance values, which quantify the similarities between groups. The software tpsDig, tpsRegr, and tpsRelw were used for geometric morphometric analyses.

A t-test was conducted to compare the mean wing size between sexes. Multivariate Analysis of Variance (MANOVA) was performed to examine the effect of sex on wing shape variables based on mean differences between groups. Statistical differences in mean wing size between flying and flightless insects, as well as between sexes, were tested using Welch's analysis of variance (Welch's ANOVA). Multivariate Analysis of Covariance (MANCOVA) was conducted to assess how wing size might explain shape variation. Statistical analyses were performed using JMP and PADwin software.

As expected, the t-test revealed significant differences in mean wing size between females and males, with females exhibiting larger wings than males (p = 0.005). Conversely, MANOVA results indicated that wing shape did not significantly differ between sexes (p = 0.0614). In this analysis, sex was treated as the independent variable, while shape variables were the dependent variables.

The selection of six species aimed to evaluate differences in wing shape and size between flying and non-flying insects within the genus *Triatoma*. By grouping species of the same genus, this study sought to determine, in a broader context, whether wing shape and size influence dispersal. To achieve this, geometric morphometric analysis was applied to measure these differences, ensuring that species-specific variations were excluded, thus allowing conclusions to be extrapolated at the genus level.

When considering both sexes together, significant differences in mean wing size were observed between flying and non-flying insects, according to Welch's ANOVA (p = 0.2767) (Figure 2).

**FIGURE 2:**
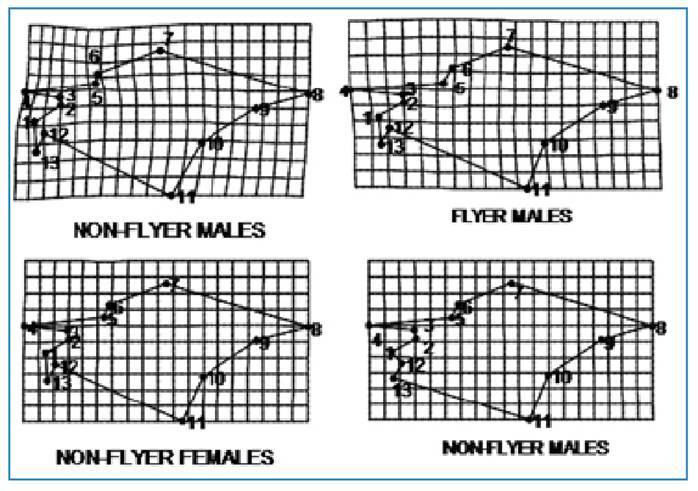
General deformations in wing shape between flying and non-flying insects relative to a consensus configuration. Deformations were estimated based on the first principal component axis (also known as the first relative warping) derived from a principal component analysis of shape variables. Landmark displacements (amplified threefold) correspond to deformation grids and illustrate the direction of morphological change. **NF-F:** non-flying females; **F-F:** flying females; **NF-M:** non-flying males; **F-M:** flying males.

Meanwhile, female wing size showed significant differences (p = 0.0008), whereas male wing size did not (p = 0.2767) (Figure 2).

Analysis of wing shape also revealed significant differences between flying and flightless insects across both sexes, as demonstrated by MANOVA results (p = 7.8995 × 10⁻⁶).

In this analysis, flight ability (flying vs. non-flying) was the independent variable, while shape variables were treated as dependent variables.

For each sex, non-parametric analyses were conducted due to small sample sizes. Mahalanobis distances were calculated from the wing shape variables, and permutation tests were performed to generate a null hypothesis stating that the Mahalanobis distance observed in the real data was not significantly different from the distances derived from 1,000 random iterations. The results indicated that Mahalanobis distances significantly distinguished the wing shapes of flying and flightless females and males.

MANCOVA was performed using wing size as the independent variable and wing shape variables as dependent variables. The results indicated a significant association between size and shape (p = 1.68 × 10⁻⁷).

Additionally, shape variation within the two groups exhibited the same relationship with the size variable. This was observed in a MANCOVA where size and group classification (flying vs. non-flying) were used as independent variables, and shape variables were used as dependent variables (p = 0.2060). This finding suggests that the allometric growth patterns of the wings were similar in both groups. The same relationship was observed when considering both sexes together and when analyzing females separately (p = 0.0016).

For females, the relationship between size and shape remained consistent between flying and non-flying groups (p = 0.3694). However, in males, no significant allometric growth pattern was detected in either group (p = 0.1627).

Differences between non-flying and flying insects were primarily defined by localized deformations at landmarks 3 to 5, which are situated at the border between the leathery and membranous regions of the wings. Non-flying insects exhibited a reduction in these landmarks, whereas flying insects displayed an expansion at the same points. These differences were more pronounced in males than in females (Figure 2).

Triatomine flight activity in natural environments remains poorly understood due to ethical and logistical constraints that limit the feasibility of conducting field experiments. Laboratory studies provide an alternative approach to expanding knowledge of this key dispersal mechanism, which influences the geographic distribution of species and, consequently, the spread of Chagas disease^11^ ^-^ ^15^.

Wings play a critical role in locomotion and dispersal, particularly in the search for shelter and hosts, thereby facilitating the colonization of new environments. They are two-dimensional structures, and the intersections of wing veins serve as taxonomically significant anatomical landmarks. By analyzing wing shape variation, it is possible to establish relationships between genetic and environmental factors, describe new species, and identify potential sources of reinfestation^11^ ^,^ ^14^ ^,^ ^15^.

Correlations between species were not conducted primarily due to the low sample size. However, the primary focus of this study was to identify patterns at the genus level, given the diversity of these vectors. Although intraspecific variations occur within this genus, identifying shared patterns across species can aid in recognizing morphological traits that may be attributed to the genus.

The reduction in wing size or complete wing loss typically occurs in one sex. In females, this phenomenon is associated with the allocation of energy toward mating and oviposition, while in males, it is linked to the formation of colonies, where dispersal for mating is unnecessary^8^. When analyzing both sexes together, we observed that mean wing size differed significantly between flying and non-flying insects. However, when analyzing males and females separately, only females exhibited a significant difference in wing size between flying and non-flying individuals.

Studies using light traps containing malnourished or starving triatomines suggest that flight ability may be impaired by poor nutritional status^11^. Several hypotheses have been proposed regarding differences in flight activity between male and female triatomines. Females initiate dispersal by flight earlier than males and travel greater distances, likely as a behavioral strategy to locate food sources necessary for egg maturation following mating. In contrast, males are physiologically prepared to mate immediately after the imaginal molt, regardless of their nutritional status^12^ ^,^ ^13^.

The absence of flight musculature and reduction in wing size are key characteristics that distinguish non-migratory species from those with migratory potential. While maintaining flight capability incurs a high metabolic cost, the development of flight structures, such as wings and flight muscles, is relatively less energy-intensive. As a result, natural selection tends to eliminate energy expenditures associated with flight when dispersal is not necessary^14^.

The ability to fly represents an evolutionary milestone in insect history, enabling the colonization of new environments. Among Triatominae, flight capability varies between species and individuals, reflecting ecological adaptations and evolutionary pressures^8^.

This study supports the existence of intraspecific sexual dimorphism, highlighting the importance of identifying species and individuals with the potential for active dispersal by flight. Flight enabled dispersal facilitates the invasion of new environments, and by identifying species with high dispersal potential, it is possible to estimate the geographic extent of transmission risk and prioritize surveillance efforts in high-risk areas.

Changes in wing shape and size can directly influence flight performance. Smaller wings may reduce flight range or efficiency.

While asymmetrical wings can impair flight stability. Species that are highly adapted to specific habitats, such as indoor environments, may develop smaller wings. These morphological changes can determine whether a species actively disperses by flight or remains more restricted to a terrestrial environment. This suggests a causal relationship between wing morphology and dispersal behavior.

In the present study, geometric morphometric analysis successfully identified distinct wing shape patterns in flying and non-flying specimens of six *Triatoma* species, detecting differences that may help identify species with the greatest capacity for active dispersal.
